# Sampling for malaria molecular surveillance

**DOI:** 10.1016/j.pt.2023.08.007

**Published:** 2023-11

**Authors:** Alfredo Mayor, Deus S. Ishengoma, Joshua L. Proctor, Robert Verity

**Affiliations:** 1 ISGlobal, Hospital Clínic – Universitat de Barcelona, Barcelona, Spain; 2 Centro de Investigação em Saúde de Manhiça (CISM), Maputo, Mozambique; 3 Department of Physiologic Sciences, Faculty of Medicine, Universidade Eduardo Mondlane, Maputo, Mozambique; 4 National Institute for Medical Research (NIMR), Dar es Salaam, Tanzania; 5 Faculty of Pharmaceutical Sciences, Monash University, Melbourne, VIC, Australia; 6 Harvard T.H. Chan School of Public Health, Harvard University, Boston, MA, USA; 7 Institute for Disease Modeling in Global Health, Bill and Melinda Gates Foundation, Seattle, WA, USA; 8 MRC Centre for Global Infectious Disease Analysis, Imperial College, London, UK

**Keywords:** malaria, genomics, molecular surveillance, sampling, antimalarial drug resistance, malaria transmission

## Abstract

Strategic use of *Plasmodium falciparum* genetic variation has great potential to inform public health actions for malaria control and elimination. Malaria molecular surveillance (MMS) begins with a strategy to identify and collect parasite samples, guided by public-health priorities. In this review we discuss sampling design practices for MMS and point out epidemiological, biological, and statistical factors that need to be considered. We present examples for different use cases, including detecting emergence and spread of rare variants, establishing transmission sources and inferring changes in malaria transmission intensity. This review will potentially guide the collection of samples and data, serve as a starting point for further methodological innovation, and enhance utilization of MMS to support malaria elimination.

## Highlights

Evidence generated from malaria molecular surveillance (MMS) can inform decision making by malaria control programs, that is, early detection of genetic variants that confer partial resistance to malaria therapy drugs (*pfkeltch13*), identifying transmission sources, and characterizing malaria transmission dynamics.
Clear and accessible guidance for appropriately designing and powering MMS use-cases does not yet exist.
Designing MMS and studies for each use-case requires defining the target population, the sampling technique, the sampling period and frequency, and the appropriate sample size required to draw valid and unbiassed conclusions.
Sample size calculations require defining assumptions about the expected prevalence of the outcome of interest, its heterogeneity in the population, the desired confidence, and the design effect in cluster-based sampling.

## Teaser

Strategic use of *Plasmodium falciparum* genetic variation has great potential to inform public health actions for malaria control and elimination. Malaria molecular surveillance (MMS) begins with a strategy to identify and collect parasite samples, guided by public-health priorities. In this review we discuss sampling design practices for MMS and point out epidemiological, biological, and statistical factors that need to be considered. We present examples for different use cases, including detecting emergence and spread of rare variants, establishing transmission sources and inferring in malaria transmission intensity. This review will potentially guide the collection of samples and data, serve as a starting point for further methodological innovation, and enhance utilization of MMS to support malaria elimination.

## The importance of adequate sampling for MMS

**Malaria molecular surveillance (MMS)** (see Glossary) is increasingly becoming a primary goal of public health surveillance efforts by National Malaria Control Programmes (NMCPs) in Africa [1., 2., 3.]. Genomic data provide a fundamentally different type of information that can supplement traditional metrics like prevalence and incidence, which, when leveraged correctly, can answer key questions relevant for malaria control/elimination. In recent years, *Plasmodium* genomic data have been used to determine the evolution and spread of **molecular markers for drug-resistant malaria** [4,5], measure connectivity and importation [6., 7., 8., 9.], estimate changes in transmission intensity [1., 2., 3.,10., 11., 12.], quantify variation in vaccine effectiveness [13], and disentangle relapse from reinfection events [14] among other applications.

The first step in any MMS study is sample collection, which is generally followed by sequencing, bioinformatic analysis, statistical analysis, reporting, and communication with public health agencies to inform data-driven responses. **Sampling** approaches and sample size calculations are a critical step in this pipeline, however, there is a lack of clear and accessible guidance for appropriately designing and powering MMS studies. Many MMS efforts to date have been conducted using convenience samples collected at health facilities, schools and antenatal care clinics [15., 16., 17., 18.], or in the context of studies designed for other purposes (malaria indicator or demographic and health surveys). As these convenience-based approaches often contain **sampling biases** and cannot be treated as truly random samples [19], molecular data might not be representative of the temporal and geographic heterogeneity of the marker of interests (i.e., drug resistance markers), and likely do not represent their true spatially dependent distribution [20]. Another major issue is statistical power and choosing the appropriate sample size. An insufficient sample size may lead to large uncertainty in estimated outcomes and a low probability of demonstrating the desired difference, while on the other end of the spectrum a very large sample size may reach diminishing returns in terms of statistical power. Both situations have negative implications in terms of cost, logistics, and the ethics of recruiting individuals for only marginal gains or for studies that are unlikely to succeed. There is also an opportunity cost to running under- or over-powered studies, which potentially take resources away from other important questions and delay the dissemination of key findings for public health action.

Without clear and standardized guidance, it is difficult to design studies in a way that maximizes the chance of success. Moreover, harmonization of sampling approaches can increase the comparability of results and inform regional approaches in a more systematic way. Here, we explore this issue by reviewing sampling approaches used for malaria surveillance, including factors (epidemiological, biological, and statistical) that need to be considered for robust study design and by providing specific examples for various MMS use cases. While we focus on the predominant *P. falciparum* species in Africa and next generation sequencing, principals of MMS and design concepts addressed here are expected to be similar for the surveillance of non-falciparum species and other molecular approaches [21].

## First step: the purpose of sequencing efforts

**Figure 1:**
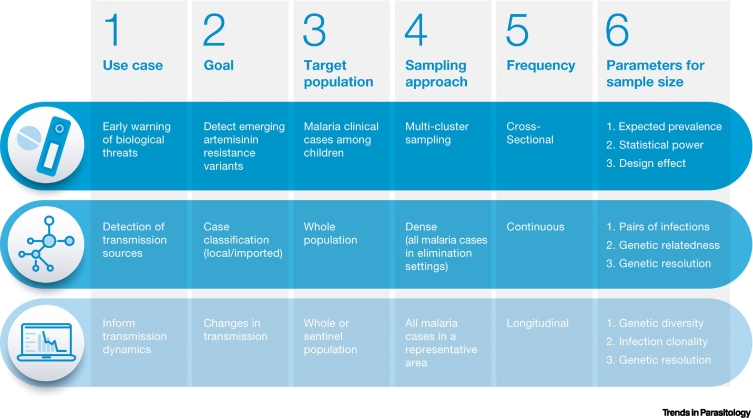
Steps for designing a malaria molecular surveillance (MMS) approach. To draw valid conclusions from MMS efforts, it is key to carefully decide how to select a sample that is representative of the target population. The surveillance purpose, and therefore the programmatic action expected from those efforts, will inform the relevant population to be sampled (which should be driven by the intervention target), the sampling method and the periodicity. All these parameters, which should be specific to the pre-defined population of interest as well as reflective of the logistical and biological sources of bias at the time of sampling, together with assumptions about the distribution of the marker of interest in the study population, need to be considered to calculate the appropriate sample size. Here we exemplify the different steps for three specific surveillance objectives: the detection of emerging variants of concern (such as mutations in *pfkelch13* associated with artemisinin resistance), the classification of cases as local or imported, and the detection of changes in transmission.

Designing a pathogen surveillance system must begin with identifying the primary purpose or key questions to be answered [11], as different goals require different sampling approaches and sample sizes in order to obtain reliable results. For MMS these goals may include (Figure 1):

### Early warning of biological threats to ensure a timely response

One of the primary use-cases for MMS is the detection of genetic **variants of concern** (**VOC**) that can compromise available therapeutic, preventive, and diagnostic tools [13,22., 23., 24., 25., 26., 27., 28.], and therefore constitute **biological threats** of surveillance interest. Broadly speaking, there are three main types of surveillance goals: (i) early detection of emerging VOC, for example *pfkelch13* mutations that confer partial resistance to front-line artemisinin-based drugs [4,29,30]; (ii) measuring the prevalence of already existing variants and tracking their spread over space and time, as is the case for mutations in the *pfdhps*, *pfdhfr*, and *pfcrt* genes that confer resistance to sulfadoxine and pyrimethamine (used for **preventive chemotherapy**) and chloroquine respectively [22,31], as well as *pfcsp* haplotypes that may increase in frequency after the implementation of RTS,S/AS01 vaccine [13,28], potentially leading to vaccine breakthrough infections; and (iii) comparing the prevalence of VOC against thresholds that motivate the immediate need for changes in control practices, for example, deletions of the *P. falciparum* histidine-rich protein 2 and 3 (*pfhrp2/3*) gene [24., 25., 26., 27.] causing false-negative rapid diagnostic test (RDT) results that, when present at frequencies above a defined threshold (currently 5%), warrant a change in RDTs [23].

### Characterize transmission sources

A better understanding of key drivers of malaria transmission can inform where and how to implement interventions for maximizing impact through tailored approaches. Genomic tools can increase the power to identify imported cases in elimination settings and their impact on local transmission [6,7], confirm linkages between locally transmitted cases in outbreak investigations [32,33], identify transmission sources [32,34], and determine cross-border connectivity of parasites for a coordinated response between countries. While existing surveillance measures (e.g., parasite rate, case incidence, and reported travel histories) are fundamental to answering some of these questions, they are often limited by consistency and accuracy, particularly in areas with highly mobile and migrant populations.

### Inform transmission dynamics

The ability to accurately estimate the intensity and trends in transmission from malaria genomic surveillance data can provide NMCPs with an important and complementary set of indicators alongside routine surveillance data to stratify regions according to transmission characteristics and monitor the impact of interventions [1., 2., 3.,^10^,^11^]. **Genetic diversity** measures such as the **complexity of infection** have been suggested as a proxy for local transmission intensity [1,12]. These genetic measures may be especially important in near-elimination settings to identify point outbreaks versus sustained local transmission [35]. However, more evidence is needed to validate genomic data with respect to traditional epidemiology.

## Second step: the target population

Once the relevant question is identified, the next step is to define the population from which to draw conclusions (Figure 1). The sampling population will be dependent on the programmatic action expected from surveillance efforts: symptomatic individuals most affected by malaria disease (i.e., children) if the surveillance purpose is to ensure appropriate diagnostic and treatment of clinical cases, asymptomatic children or pregnant women if the purpose is to guide chemoprotective approaches [36], and all infections emerging in a given area, irrespectively of age, when the aim is to inform the contribution of imported cases to local transmission. However, the accessible population (the portion of the population to which the surveillance system has reasonable access) may not always coincide with the ideal target population. For example, sampling asymptomatic individuals tends to require logistically complex and costly household surveys, and low parasite densities in these infections [37] may reduce the success of sequencing efforts [38]. Instead, clinical cases or pregnant women at first antenatal care visit [18,39] might be targeted for sustainable MMS approaches as long as the potential bias can be quantified or acknowledged. Also, targeting adults for surveillance of *pfhrp2* deletions may impose a high cost in term of secondary lactate dehydrogenase-based RDTs [23] due to the lower risk of detectable *P. falciparum* infections compared to children. It is important to note that any prospective study design that is collecting private and confidential data from individuals should adhere to the ethical standards required by the institutions and countries to ensure consent and data privacy. Also, special attention is required to select the appropriate samples for subsequent molecular analysis (such as whole blood, dried blood spot, or discarded RDTs), as this decision may impact the feasibility of the sampling approach (blood drawing and storage) and the yield of the molecular assay (which will be dependent on the amount of blood from which parasite DNA will be extracted). Further studies are needed to assess the similarity of parasites collected from different human subgroups, the impact of immunity and comorbidities (e.g., HIV infection) [40] on parasite genetic patterns, and the effect that difference sample types can have on the performance of the molecular assays.

## Third step: the sampling approach

With the target population defined, the next question is how to sample from this population in an unbiased manner. The most straightforward approach from a purely statistical perspective is **simple random sampling**, a **probability sampling** where every member of the target population has the same chance of being included in the sample. However, this approach is almost always infeasible when performing surveillance over large geographic areas. For example, a national-level study under simple random sampling would involve choosing individuals at random from the entire country. From a logistical perspective it makes more sense to sample multiple individuals from the same location; however, previous reports have found high levels of spatial heterogeneity in markers of drug resistance down to the subnational level [4,20,41,42], meaning that a single spatial location may be at risk of grossly over- or under-estimating prevalence. For this reason, **cluster sampling** is often used as a middle ground between these two approaches. In cluster sampling pre-existing units (clusters) such as health clinics or households are randomly selected, typically followed by sampling individuals at random within each cluster (multistage sampling). For this approach, a systematic random sample of clusters should be selected from a complete list of all available (i.e., all health facilities in a province or region) with probabilities proportional to the estimated numbers of individuals that they service (probability proportional to size sampling) [23]. The composition of the clusters and how well they represent the larger population determines the validity of the results, while the number of clusters determines the precision and the length of time needed to meet enrolment targets. As cluster sampling usually requires many resources and intensive logistic planning, **sentinel site sampling** at predefined health facilities may represent a pragmatic alternative when a truly representative sample of the whole of the territory is difficult to obtain [43,44]. Although problematic from a statistical perspective, as it generates the possibility of bias, a careful selection based on geographical size, population distribution and density, and malaria epidemiology may effectively answer the surveillance objectives and avoid suboptimal use of resources or even inaccurate results [45]. Finally, nonrandom or purposive (**nonprobability**) sampling can be used for investigating particular cases of public health interesti. Similarly, targeted [46] or **risk-based sampling** [47], which considers clinical (e.g., immunocompromised patients, severe malaria cases), geographic (e.g., outbreak investigations or incoming travelers returning from specific destination) or demographic risk factors might be used to increase the probability of finding certain variants of interest.

## Fourth step: the sampling period and frequency

In addition to the size and distribution of the population surveyed, it is important to decide when and with what periodicity the surveillance will be implemented. Malaria seasonality is an important consideration in these decisions, especially for regions with highly seasonal transmission, multiple peaks within a season, and interventions deployed across the season. It will determine the time required to achieve the target sample size and may affect genetic outcomes that are of interest in some cases, such as genetic complexity or the fraction of polygenomic infections [48,49]. If the purpose is to quantify the frequencies of drug-resistance alleles in the parasite population, then it would be necessary to sample parasites before treatment, or a considerable time after chemopreventive interventions to avoid the short-term temporal changes in allele frequencies while antimalarials are present in blood [50,51]. Other than the timing of the sampling, the frequency needs to be decided depending on the surveillance purpose. Surveys range from cross-sectional, if the aim is to gather information on a population at a single point in time [52], to longitudinal, if the aim is to detect temporal changes in the outcome of interest [53]. More complex designs are also available, such as pseudo-longitudinal sampling in which a cross-sectional design is applied repeatedly but sampling differently in each wave [54]. Sampling frequency will influence how sample size is defined and what targets must be specified to calculate the appropriate sample size.

## Fifth step: the sample size

Once we have a firm idea of the sampling approach and frequency, we can begin to ask specific questions about the minimum number of participants required to arrive at statistically sound conclusions. Sample size calculation is a mature field in its own right, complete with its own language and terminology (Table 1). We give some examples for MMS specific use cases here.

**Table 1:** Parameters to be considered in the calculation of a sample size

Parameter	Description
Population size	• Total population size from which the sample will be drawn and about which researchers will make conclusions. • If the target population is small (less than 10 times the sample size) then a **finite population correction** may be required.
Expected prevalence	• Information regarding expected prevalence should be obtained from the literature, from expert knowledge or by carrying out a pilot-study. • When this information is not available, the value that maximizes sample size can be used (usually 50% prevalence).
Intracluster correlation coefficient (ICC)	• For a clustered study design, the level to which individuals from the same cluster have correlated outcomes, due to (i) similar behaviors and risk factors, (ii) the cluster itself introducing correlations, or (iii) the process of disease transmission introducing correlations. • Leads to diminishing returns when sampling more people from the same cluster, and favors instead larger numbers of clusters. • Reasonable estimates of the ICC can be obtained from the literature or from expert knowledge. Pilot studies will often be underpowered to estimate the ICC.
Design effect ($D_{\mathrm{eff}}$)	• Ratio of the variance of a statistic with a complex sample design to the variance under simple random sampling. Larger values represent less efficient designs, with 1 representing perfect efficiency. • A large design effect needs to be compensated by an increase in sample size. • In general, a design effect of 1.5 or 2 is considered reasonable. However, much larger values are plausible in practice, and values should be tailored to the study where possible. • For clustered surveys, the design effect can be calculated from the ICC through the formula $D_{\mathrm{eff}}=1+\left(n-1\right)\times \mathrm{ICC}$, where n is the per cluster sample size.
Significance level (Alpha)	• A predetermined threshold that determines the strength of evidence required to reject the null hypothesis. • Represents the maximum acceptable probability of making a type I error (false positive). • A value of α=5% is often used. Smaller values need to be compensated by larger sample sizes.
Statistical power (1-Beta)	• The probability of correctly rejecting the null hypothesis when it is indeed false. • A value between 80% and 90% is usually used. • The greater the power, the larger the sample size required.
Margin of error (MOE)	• A measure of the amount of sampling error we expect in the results of our survey. • Can be used to select sample sizes in cases where no hypothesis test is being performed. • The smaller the MOE, the larger the sample size required.

### Drug and diagnostic resistance studies

From a purely statistical point of view, both drug and diagnostic resistance studies aim to do the same thing: to estimate the prevalence of a genetic marker in a population. This can be made more or less complex as we see fit, for example establishing whether prevalence is above a given threshold or comparing prevalence from one year to the next. Here, we give two examples of how statistical considerations can guide study design for cross-sectional prevalence surveys.

#### Testing for a single copy of a rare variant

Imagine that we are planning to conduct a study to establish if the *pfkelch13* mutation R561H [4] is present in our target population (but not to estimate its prevalence). We can phrase this as a hypothesis test in which the null hypothesis is that the prevalence of the mutation is 0%. We will reject this claim if we see a single copy of the R561H mutation in our sample. The question becomes: what is the probability of rejecting the null hypothesis? Using the formula in Box 1 (Question A), we find that n=161 samples are required to reach 80% power if the variant is present at 1% prevalence. This is a well powered study, but it is important to recognize that we still have a 20% chance of seeing no variants and therefore failing to reject the null hypothesis. Unfortunately, there is no way around this, as to be 100% confident we would need to survey the entire population. The aim of power analysis is not to achieve 100% power, rather it provides a framework that can be used to relate the chance of success of an experiment to other factors that cannot be controlled. Importantly, it requires assuming a particular prevalence in order to calculate sample size. There are no hard rules on how to do this, and the best approach is often to be pragmatic. For example, we can ask whether a prevalence of 0.1% *pfkelch13* mutations would actually result in a programmatic change in the way drugs are delivered. If the answer is no, then arguably we do not need to be powered to detect this low-level prevalence. Conversely, a prevalence of 20% may by unlikely a priori as it would already be apparent in clinical failure rates, and so we can safely rule out such high values. When comparing across assumptions, it can also be useful to consult tables such as Table I in Box 1, which gives minimum sample sizes across a range of prevalence assumptions.

Box 1 **Sample size estimates for cross-sectional prevalence surveys**

**Question A: is a variant present in the population?**

Assume that the true prevalence of a variant in the population is given by p, and we intend to take a cross-sectional purely random sample of size n. If sampling is independent, then the probability that all n samples are negative for the variant is given by the formula:

[I] $$\mathrm{Pr}\left(\text{Zero variants found in sample of size}\;n\right)={\left(1-p\right)}^{n}$$

We can rephrase this in terms of statistical power, defined as the probability of correctly rejecting the null hypothesis. Here, power would be the probability of correctly concluding that the variant is present in the population, which is the inverse of the formula above:

[II] $$\text{Power}=1-{\left(1-p\right)}^{n}$$

For example, if the prevalence of the variant is 1% and we take a sample of size n=50 then our study has a power of 39%, meaning we are more likely than not to fail to disprove the null hypothesis, that is, to come to a false-negative conclusion. We can rearrange equation II to give us the sample size required to achieve a target power:

[III] $$n=\frac{\log\left(1-\text{Power}\right)}{\log\left(1-p\right)}$$

Table I uses this formula to calculate the minimum sample size for a range of prevalence assumptions and target power levels. Note, this calculation applies to the question of detecting a variant in a specific site only; if the aim is to make conclusions at a higher geographic level (e.g., province) then a multi-cluster approach is required (see later).

**Table I:** Minimum sample size required to detect at least one copy of a variant for a range of prevalence and power assumptions

Power	Prevalence of variant in population						
	0.1%	0.2%	0.5%	1%	2%	5%	10%
70%	1204	602	241	120	60	24	12
80%	1609	804	322	161	80	32	16
90%	2302	1151	460	230	114	45	22

**Question B: what is the prevalence of a variant in the population?**

Assume that we are conducting a multicluster survey to estimate the prevalence of a variant at the province level. The study consists of c clusters each containing n samples. Our analysis plan is to estimate prevalence in each cluster and then to take the mean of these values to obtain the overall estimate $\hat{p}$ for the province. The expected margin of error (d) of this estimator is given by:

[IV] $$d=z\sqrt{\frac{p\left(1-p\right)}{\mathrm{nc}}\left(1+\left(n-1\right)r\right)}$$

where p is the true prevalence at the province level, r is intra-cluster correlation coefficient and z is the critical value of the normal distribution (z=1.96 for a two-sided interval at 95% confidence). In other words, if the true prevalence is p then we expect $\hat{p}$ to fall within p±d around 95% of the time. We can rephrase this as a sample size calculation by rearranging equation IV in terms of n:

[V] $$n=\frac{z^{2}p\left(1-p\right)(1-r)}{cd^{2}-z^{2}p\left(1-p\right)r}$$

Using this formula, we can decide on a tolerable margin of error (d) and then choose the sample size that achieves this precision. Table II gives the sample sizes required to achieve a margin of error of d=5%. Values give the sample size per cluster, which can be multiplied by the number of clusters to obtain the total sample size of the study.

**Table II:** Minimum sample size required per cluster to estimate prevalence to within a margin of error of 5% in a multcluster survey

Number of clusters	Prevalence of variant in population								
	10%	20%	30%	40%	50%	60%	70%	80%	90%
Intra-cluster correlation of 0.005 (low)									
1	447	–	–	–	–	–	–	–	447
2	106	318	831	2352	4827	2352	831	318	106
3	60	139	232	318	355	318	232	139	60
4	42	89	135	171	184	171	135	89	42
5	32	65	95	117	125	117	95	65	32
6	26	52	74	89	94	89	74	52	26
7	22	43	60	72	76	72	60	43	22
8	19	37	51	60	63	60	51	37	19
9	17	32	44	52	54	52	44	32	17
10	15	28	39	45	48	45	39	28	15
Intra-cluster correlation of 0.02 (moderate)									
1	–	–	–	–	–	–	–	–	–
2	–	–	–	–	–	–	–	–	–
3	580	–	–	–	–	–	–	–	580
4	110	–	–	–	–	–	–	–	110
5	61	2912	–	–	–	–	–	2912	61
6	42	223	–	–	–	–	–	223	42
7	33	116	580	–	–	–	580	116	33
8	26	79	205	580	1189	580	205	79	26
9	22	60	125	223	286	223	125	60	22
10	19	48	90	138	163	138	90	48	19

Blank cells indicate that the required precision cannot be reached (in which case equation V returns a negative value).

Alt-text: Box 1

#### Estimating prevalence of a variant within a margin of error

In some cases, we may be confident that a variant is present but want to estimate its prevalence. We can still make sample size recommendations, this time based on precision arguments rather than statistical power. For example, imagine that we are planning a large, multicluster survey to estimate the prevalence of the *pfdhps* K540E mutation at the province or regional level, combining information over multiple health facilities (clusters). As before, we have to start by assuming a particular prevalence of the variant when performing our sample size calculation. This time we also need to specify the level of variation between clusters, which may be quite large if we expect a high degree of spatial heterogeneity in our study region. In our case, let us assume that the prevalence is 30% at the province or regional level and that the intra-cluster correlation is 0.005. We can then use the formula in Box 1 (Question B) to calculate the sample size required in each cluster to estimate prevalence to within a defined margin of error. For example, if we recruit 10 clusters and want to estimate prevalence to within ±5% margin of error then we need 39 samples per cluster (390 total). Table II in Box 1 gives these sample sizes for a range of assumptions. Notice that the total sample size (the per-cluster value multiplied by the number of clusters) decreases as the number of clusters increases, meaning that it is more statistically efficient to survey many clusters than it is to intensively collect samples from a few. This is due to intra-cluster correlation, which means we reach diminishing returns when we continue to sample from the same sub-population [55] (Table 1). Also notice that sample sizes for large numbers of clusters are more robust to different assumptions about intra-cluster correlation, which is another argument for recruiting large numbers of clusters where possible.

### Importation and spatial connectivity

**Genetic relatedness**-based approaches have recently been shown to be a powerful tool for identifying geographic regions that are linked by transmission [8]. The raw data for these methods ranges from biallelic single-nucleotide polymorphism (SNP) data as produced in whole-genome sequencing [56], to unlinked multiallelic data such as microsatellites [57]. However, analytical tools to translate genetic connectivity to demographic connectivity (e.g., the number of parasites imported from one location to the other) are still missing. This is a major problem in most areas of sub-Saharan Africa where human connectivity and parasite flow occurs between genetically intermixed populations. Tracking of infections using this approach is based on the genetic similarity between the ensemble of parasites present in each of a pair of infections representing a potential transmission event [9]. This is a nascent area of genomic surveillance, and the tools do not currently exist for performing exact power and sample size calculations for this use-case. However, some basic arguments can be useful when considering study design, which include the type of sequencing and the overall population diversity (Box 2). For a sample of size *n* there are *n*-choose-two pairs, which is equal to ½ × *n* × (*n* – 1). So, for a sample of size of 10 there are 45 pairs, but for a sample of size of 20 there are 190 pairs. By doubling the sample size, we more than quadruple the number of possible pairwise links we are exploring. A similar argument can be made when comparing between distinct locations. Now, if we have *n*_*1*_ samples from the first location and *n*_*2*_ samples from the second, then the number of possible links between areas is $n_{1}\times n_{2}$. Going from a sample size of 10 in each location to 20 increases the number of possible links from 100 to 400. For these reasons, **deep sampling** in a local area is recommended when the aim is to detect pairs of samples that form recent transmission chains.

Box 2 **Sample size considerations for parasite connectivity**

A major factor in our ability to detect connectivity is what proportion of sample pairs in the population carry highly related parasites (Figure I). This is influenced by multiple factors, but a very important one is transmission intensity. In high-transmission settings, overall relatedness will tend to be lower due to increased recombination, and for this reason, the ability to infer networks of transmission from genetic data tends to be highest at low transmission. At very low transmission, however, samples become scarce to reach the desired sample size and increasingly clonal, meaning genetic data ceases to be informative of transmission links.

The sequencing approach also influences the ability to detect this high relatedness from the genetic data and therefore the power of the study. The most powerful methods are based on the use of **identity by descent** (**IBD**), a probabilistic measure of the fraction of the genome that a pair of parasites inherited from a recent common ancestor. For **targeted amplicon sequencing** methods, our ability to reliably detect IBD is highest when using a large number of loci, and when they are diverse (ideally multiallelic) and distributed throughout the genome. Accordingly, previous studies [56] have found that larger sample sizes are required when fewer loci are included in the analysis [9]. When looking for spatial trends in relatedness, it has been estimated that for whole-genome data approximately 147 single-infection samples were required, whereas for samples sequenced at 93 single-nucleotide polymorphisms (SNPs) or at 24 SNPs, 222 and 344 single-infection samples, respectively, would have been required for the same power [8]. Similarly, previous studies [58] and simulated data [56] have shown a rapidly decreasing accuracy in the expected IBD fraction with fewer SNPs. Moreover, 24 and 93 SNP barcodes have not been recommended for IBD-based analyses of individual parasite sample pairs due to large expected error in that application [56].

**Figure I:**
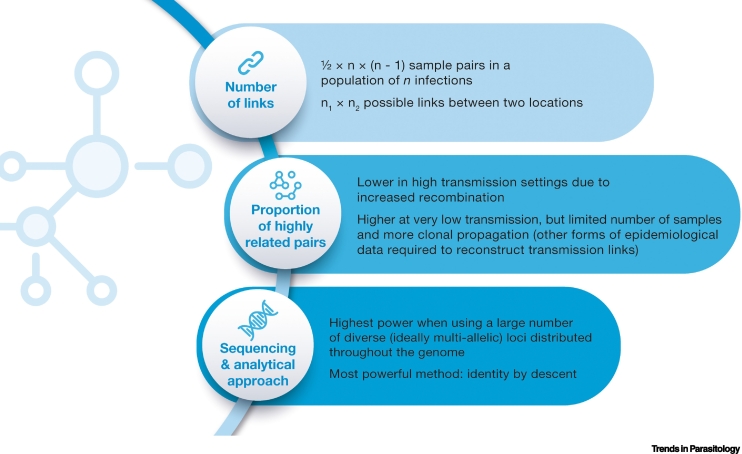
Factors that determine the ability to detect parasite connectivity.

Alt-text: Box 2

### Trends in malaria transmission

Several studies highlight the associations between malaria burden and parasite genetic measures, such as the fraction of samples that are classified as having multiple infections, the proportion of unique strains, and the amount of persistent **clonal propagation** in a population [1,2,58., 59., 60., 61., 62.]. This empirical evidence supports the underlying hypothesis that the biological mechanisms of **superinfection** and **cotransmission** are linked to different transmission intensities and are visible in measures of within-host and population-level parasite diversity. However, estimating a single sample size to assess transmission characteristics across a broad set of geographies remains an open challenge. One of the difficulties stems from the complex and dynamic interplay between the number of samples collected each year, at what time the samples are collected during that year [59], the genetic resolution of the sequencing technology [63,64], the underlying genetic population diversity [64], the modes and magnitude of transmission in the region, especially in highly seasonal regions with an unknown amount of imported infections [60,64], and non-linear, region-specific relationships between genetic and transmission measures [2,65]. Previous studies exhibit limitations associated with a convenience sampling approach [58,59], and an incomplete characterization of local transmission due to a lack of prevalence or entomological inoculation rate measurements [58] and varying levels of health seeking [66]. Mathematical malaria genomic models have been developed, incorporating biological mechanisms such as superinfection and co-transmission at varying levels of complexity, to help mitigate some of these challenges and to assess the link between specific genetic measures and a true modeled transmission rate. Here, models can be used as a tool to investigate the conditions under which certain observations could arise and generate sample size estimates for different use cases [12,58,59] (Box 3). However, additional evidence and investigations are required to systematically link parasite genetic data and diversity measures to more traditional measurements of parasite transmission [10,11].

Box 3 **Sampling factors to infer levels and changes in malaria transmission**

The use of MMS data to understand levels and changes in malaria transmission is viewed as confirmatory, or as an augmentation to traditional epidemiologic data, either because data is sparse due to limited surveillance infrastructure or where traditional metrics are insensitive to relevant changes in transmission [58]. Previous studies have identified associations of population diversity and routine measures of transmission using relatively small sets of single nucleotide polymorphisms (SNPs), typically between 24 and 100 SNPs, with approximately 100 or more samples per year [58,60., 61., 62.]. Increasing the genetic resolution to 100 SNPs can provide more dynamic range for population measures such as identifying higher resolution connectivity between samples in low-intensity and low-diversity regions, but also increases fidelity of within-host diversity measures, such as measures of heterozygosity and estimates of complexity of infection that have been linked to changes in transmission [60]. Watson *et al*. [12] estimated that no more than 350 samples are required to detect a 20% decrease in malaria prevalence by nucleic acid amplification tests over five years for every genetic measure considered; however, for smaller decreases or lower starting prevalence, sample sizes need to be increased and genetic measures may not be equally predictive [12]. Moreover, the ability to resolve within-host diversity measures becomes more challenging as the intensity of transmission and the genetic complexity of infections increase in the population [60,61]. In these contexts, either a broader coverage of the parasite genome or next-generation sequencing of amplicons may be required from a similar number of samples. Lower intensity regions may require more samples and higher resolution genetic data to detect declines in transmission and fragmentation of transmission chains.

What should a gold standard prospective study consider? The design of prospective studies to assess the link between genetic features and transmission should leverage standard measurements of transmission including prevalence, incidence, entomological inoculation rates, health seeking rates, and programmatic activity and coverage. The sample collection protocol should consider the timing of collection relative to these measurements of transmission, the transmission season, the within season transmission dynamics, intervention deployments, and across regions (Figure I). Also, studies should aim to collect standard epidemiological metadata about every individual and their household as well as information regarding any recent travel in their household, always following ethical requirements to guarantee informed consent and data privacy. A holistic approach to the design of prospective studies will be essential in generating the next wave of evidence to support this malaria genomic surveillance use-case.

**Figure I:**
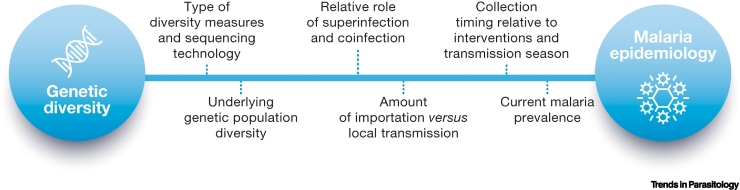
Factors that determine the ability to infer malaria transmission.

Alt-text: Box 3

## Further considerations for malaria molecular sampling

Sample sizes must always consider that a certain number of individuals and samples will not accept to participate in the study or will not produce high quality sequences due to technical issues, respectively. The study designer should estimate dropout based on past experience and inflate sample sizes accordingly. For example, if we expect 80% of samples to be of high sequence quality then sample sizes should be inflated by dividing by 0.8. This should be done in a context-specific way, for example low density infections obtained from asymptomatic individuals or the use of positive RDTs as source of parasite DNA may have lower sequencing efficiency than other infections or biological samples (e.g., dried blood spots), respectively.

Sample size estimates assume that the pool of parasites available for sequencing is a representative random sample of the total parasite population. However, if a variant of interest has differing biological or epidemiological properties (i.e., transmissibility, fitness cost or virulence) that affect parasite abundance in infected hosts (i.e., testing sensitivity and the probability of meeting sequencing quality thresholds) then calculations should be adjusted to account for the likely over or underrepresentation of this variant in the sampling pool.

In certain scenarios, surveillance efforts may encompass the simultaneous sampling of multiple markers, capturing both rare variants and those occurring at high frequencies. In this case, it becomes necessary to adequately power the study for each specific scenario and select the highest required sample size. In other cases, it may be more convenient to study the entire target population, rather than a study based on a sample of the population. This applies especially to low transmission settings, where antimalarial resistance has been hypothesized to emerge [67,68] and the number of parasite positive cases is low. In these settings, analysing all malaria cases may be crucial for the classification of malaria cases as local or imported. Therefore, sampling efforts in such settings may need to be intensified when the objective is to detect the emergence of novel resistance variants.

Samples should be selected as randomly as possible, however, true random sampling is difficult to achieve. Therefore, it is recommended to systematically test for potential sampling biases which, if detected, may be considered in the calculations to provide less biased prevalence estimates. This can be achieved by looking at descriptive statistics (i.e., demographic variables) of the sampled and represented populations [69,70] and using statistical techniques to correct these effects during study analysis [70]. To make this possible, it is essential that the sampling strategy is well-documented and taken into consideration during further data analysis and interpretation, so that bias is minimized and, where present, considered in the analysis and interpretation of data.

If the minimum sample size cannot be reached then we should not switch to a convenience-based approach, rather we should ask if we can change our study design to improve power. For example, in a study of rare variants we could focus on high-risk regions, thereby changing our prevalence assumptions and reducing the sample size required. We could also consider making an argument for a pilot study at 70% power, to be followed up later by more detailed investigation. Alternatively, we could consider reactive surveillance approaches that may require smaller sample sizes, such as lot quality assurance sampling, that allow pinpointing areas where warning signals are detected for subsequent follow-up (Box 4). Finally, sampling units (i.e., provinces) could be rotated during subsequent years to overall produce a full sampling cycle covering the whole country every 2–3 years [71]. All these designs are preferable to going ahead with a study using sample sizes based purely on logistical arguments and ignoring the statistical implications. Finally, it is important to include national malaria control programs in the design of these studies to understand their current programmatic questions, integrate their direct experience into the study design, and regularly communicate data and insights generated from the study for their decision-making. There is also a robust scientific community for sharing malaria parasite genetics data; where ethically appropriate and with the appropriate consent, actively sharing anonymized study data can help support other country programs in their study design and data collection efforts.

Box 4 **Other sampling approaches**

We have not covered all the MMS use cases and designs in this review. Several other fields, including veterinary and survey strategies to substantiate freedom from disease for a certain territory [72], are developing more sophisticated, effective and labor-efficient sampling methods that could eventually be applied for malaria genomic surveillance:

• Adaptive sampling approaches (also called response-adaptive designs) in which the procedure for selecting sites or units to include in the sample can depend on values of the variable of interest observed during the survey, *allowing a refinement of data as it becomes available* [73,74]. This sampling approaches take advantage of patterns not discovered prior to the survey, and may add samples from neighboring sites (or a larger proportion of samples in that area) whenever a variant is encountered. For example, adaptive *geostatistical designs* sequentially select sampling sites based on observations from previous iterations, and use spatial autocorrelation in the observed data to improve prediction and inference [75,76].
• Continuous sampling approaches that inform the current estimate using a rolling window approach, with larger weight given to more recent data, which might better reflect current prevalence. This approach can reduce the number of sequences required at each time step as compared to a cross-sectional sampling design [77].
• Sequential sampling, which involves the evaluation of each sample taken from a population to see if it fits a desired conclusion and stopping further sampling as soon as there is sufficient support for the conclusion [78]. This sampling method is best used in situations where the classification of the prevalence of a condition into categories or classes (for example, high and low prevalence) provides sufficient information on which to base decisions to take specific actions [79]. One classification approach is lot quality assurance sampling (LQAS), a methodology developed in the manufacturing industry to assess the quality of a batch (lot) [80] that has been used for surveillance of drug resistant tuberculosis [81] and HIV [78].
• Xeno-monitoring of parasite molecular markers in malaria vectors [82,83], potentially allowing a timelier surveillance as mosquitoes can be collected throughout the year without a long planning phase of obtaining ethical approval.

Alt-text: Box 4

## Concluding remarks

A good sampling strategy is critical to the success of a surveillance study. However, study design and optimal sample sizes are not purely statistical considerations, as they depend on many factors including logistic, budgetary, ethical and social considerations, and these factors must be balanced when coming up with final numbers. For example, it may be statistically desirable to sample a large number of spatial clusters, but at the same time this may be infeasible from a logistical or budgetary perspective. When budgets are tight, we may be tempted to abandon statistical arguments altogether in favor of purely programmatic decision-making, however, we should be wary of this approach. A study will be subject to the same degree of randomness whether we quantify it beforehand or not, so we are always better off armed with this information. When used in conjunction with other information, and acknowledging gaps in knowledge still required to be addressed (see Outstanding questions), statistical arguments can be seen as a guide to direct us towards study designs that have a good chance of success. Critical applications of this approach in the near future include designing studies to detect the presence of mutations that confer partial resistance to Artemisinin, and to measure the changing prevalence of these mutations in space and time, and similarly for *pfhrp2/3* gene deletions to estimate prevalence of deletions relative to defined thresholds.

Outstanding questions What is the economic impact of integrating genomic sequencing into malaria surveillance, and what sample approaches can be used to minimize expenditures?

What are the prevalence thresholds for molecular markers of antimalarial resistance above which use of a drug is no longer justified?

What are the spatial and temporal scales over which parasite heterogeneity can be observed and what are the implications for MMS sampling to provide comprehensive information on the population-level prevalence of variants of concern?

Which factors (i.e., geographical size, population distribution and density, and malaria epidemiology) should be considered when selecting the number and location of sentinel sites?

What is the similarity of parasites collected from different human subgroups (symptomatic, asymptomatic, pregnant women, children, adults), as well as the impact that immunity and comorbidities (i.e., HIV infection) have on parasite genetic patterns?

What is the impact of seasonality on molecular markers of surveillance interest? Can bottlenecks that occur during low transmission season also affect the prevalence of genetic variants in the next transmission season?

Which are the relatedness and diversity key markers and analysis tools that can guide sample size calculations for assessing spatial connectivity and intensity of malaria transmission?

What is the impact of sample type on the success of MMS in addressing the use cases of interest? What is the difference in the level of genetic information generated from different sample types (e.g., dried blood spot versus a small whole-blood sample versus discarded RDTs or mosquito blood meal)?

Alt-text: Outstanding questions

## Acknowledgment and funding information

Special thanks to Julie Thwing, Abdoulaye Djimde, Daouda Ndiaye, Hanna Slater, Fitsum Tadesse, and Caitlin Bever who contributed during initial discussions about sampling strategies for malaria molecular surveillance. Thanks also to Estee Torok for encouraging and supporting this review with her constant enthusiasm. This review is based on research conducted by Alfredo Mayor (ISGlobal and CISM), Robert Verity (Imperial College), Deus S. Ishengoma (NIMR), and Joshua L Proctor (IDM), and funded in whole or in part by the 10.13039/100000865Bill & Melinda Gates Foundation (INV-019032 to A.M., INV-002202 to D.S.I., and INV-031273 to R.V.), including models and data analysis performed by the Institute for Disease Modeling at the Bill & Melinda Gates Foundation. R.V. acknowledges funding from the MRC Centre for Global Infectious Disease Analysis (reference MR/R015600/1), jointly funded by the UK 10.13039/501100000265Medical Research Council (MRC) and the UK Foreign, Commonwealth & Development Office (FCDO), under the MRC/FCDO Concordat agreement and are also part of the EDCTP2 programme supported by the 10.13039/501100000780European Union. R.V. also acknowledges funding by Community Jameel. A.M. acknowledges funding from the Departament d’Universitats i Recerca de la Generalitat de Catalunya (AGAUR; 2021 SGR 01517), the grant CEX2018-000806-S funded by 10.13039/501100004837MCIN/AEI/10.13039/501100011033, and the Generalitat de Catalunya through the CERCA Program. CISM is supported by the Government of Mozambique and the Spanish Agency for International Development (AECID). This research is part of ISGlobal’s Program on the Molecular Mechanisms of Malaria, which is partially supported by the 10.13039/100008054Fundación Ramón Areces.

## Declaration of interests

The authors declare no competing interests.

## Glossary

**Biological threats to malaria control and elimination**: antimalarial and insecticide resistance, diagnostic failure (*pfhrp2/3* gene deletions) and the emergence of invasive vector species.
**Clonal propagation**: the transmission of identical parasite strains from one infection to the next.
**Cluster sampling**: groups rather than individuals of the target population are selected at random.
**Complexity of infection**: the number of genetically distinct parasite strains coinfecting a single host.
**Cotransmission**: a single mosquito bearing multiple genetically distinct parasites bites an uninfected individual.
**Deep sampling**: a sampling strategy in which a large number of samples are taken from a small number of populations (e.g., geographic sites).
**Genetic diversity**: genetic variability present within a given population.
**Genetic relatedness**: a measure of recent shared ancestry between two individuals, ranging from zero between two unrelated individuals to one between clones.
**Finite population correction**: a term that can be added to some sample size formulae to make them more correct in cases where the population size is not vastly greater than the sample size.
**Identity by descent (IBD)**: two copies of genetic material are identical by descent if they are descended from a common ancestor with no intervening mutations.
**Malaria molecular surveillance (MMS)**: the use of molecular biology approaches (i.e., serology, genotyping, whole genome sequencing) to derive epidemiologically actionable information for malaria control and elimination.
**Molecular markers for drug-resistant malaria**: genetic changes that confer parasite resistance to drugs used to treat and prevent malaria. Molecular markers do not necessarily reflect therapeutic and chemoprevention efficacy, which also depends on factors other than intrinsic parasite susceptibility, such as patient-acquired immunity, initial parasite biomass, treatment adherence, dosing, drug quality and pharmacokinetics.
**Nonprobability sampling**: nonrandom selection based on convenience or other criteria, allowing the easy collection of data. However, not every individual has the same chance of being included, therefore increasing the risk of sampling bias.
**Preventive chemotherapy**: the use of medicines, either alone or in combination, to prevent malaria infection and its consequences.
**Probability (or random) sampling**: randomization is used instead of deliberate choice, allowing strong statistical inferences to be made about the whole group.
**Risk-based sampling**: a sampling strategy that involves identifying subpopulations at greater risk of the outcome of interest and ensuring that these are represented in a proportion greater than in the general population.
**Sampling**: the process of using a subset of a population to represent the whole population in a given area, thereby allowing valid conclusions to be drawn from the results.
**Sampling bias**: a phenomenon that occurs when a surveillance design fails to collect a representative sample of a target population, therefore limiting the generalizability and external validity.
**Sentinel site sampling**: a surveillance strategy that is conducted (at specific sites selected) based on convenience criteria.
**Simple random sampling**: a type of probability sampling in which every member of the target population has the same probability of being included in the sample.
**Superinfection**: an individual is bitten by two mosquitoes, each bearing a single parasite genotype.
**Targeted amplicon sequencing**: any sequencing technology that involves selective amplification and sequencing of specifically chosen genomic regions.
**Variants of concern (VOC)**: genetic variants of the parasite that require a programmatic reaction due to their higher risk of resistance to antimalarials, diagnostic failure or other clinical outcomes of interest.

## Resources

^i^ https://www.ecdc.europa.eu/en/publications-data/guidance-representative-and-targeted-genomic-sars-cov-2-monitoring

## References

[1] Neafsey D.E. (2021). Advances and opportunities in malaria population genomics. Nat. Rev. Genet..

[2] Tessema S.K. (2019). Applying next-generation sequencing to track falciparum malaria in sub-Saharan Africa. Malar. J..

[3] Volkman S.K. (2012). Harnessing genomics and genome biology to understand malaria biology. Nat. Rev. Genet..

[4] Uwimana A. (2020). Emergence and clonal expansion of *in vitro* artemisinin-resistant *Plasmodium falciparum* kelch13 R561H mutant parasites in Rwanda. Nat. Med..

[5] da Silva C. (2023). Targeted and whole-genome sequencing reveal a north-south divide in *P. falciparum* drug resistance markers and genetic structure in Mozambique. Commun. Biol..

[6] Chang H.H. (2019). Mapping imported malaria in Bangladesh using parasite genetic and human mobility data. Elife.

[7] Sturrock H.J.W. (2015). Tackling imported malaria: an elimination endgame. Am. J. Trop. Med. Hyg..

[8] Taylor A.R. (2017). Quantifying connectivity between local *Plasmodium falciparum* malaria parasite populations using identity by descent. PLoS Genet..

[9] Archie E.A. (2009). Infecting epidemiology with genetics: a new frontier in disease ecology. Trends Ecol. Evol..

[10] Dalmat R. (2019). Use cases for genetic epidemiology in malaria elimination. Malar. J..

[11] WHO (2019).

[12] Watson O.J. (2021). Evaluating the performance of malaria genetics for inferring changes in transmission intensity using transmission modeling. Mol. Biol. Evol..

[13] Neafsey D.E. (2015). Genetic diversity and protective efficacy of the RTS,S/AS01 malaria vaccine. N. Engl. J. Med..

[14] Rovira-Vallbona E. (2021). High proportion of genome-wide homology and increased pretreatment pvcrt levels in *Plasmodium vivax* late recurrences: a chloroquine therapeutic efficacy study. Antimicrob. Agents Chemother..

[15] Thawer S.G. (2022). The use of routine health facility data for micro-stratification of malaria risk in mainland Tanzania. Malar. J..

[16] Mitchell C.L. (2022). Evaluating malaria prevalence and land cover across varying transmission intensity in Tanzania using a cross-sectional survey of school-aged children. Malar. J..

[17] Mayor A. (2019). Targeting pregnant women for malaria surveillance. Trends Parasitol..

[18] Kitojo C. (2019). Estimating malaria burden among pregnant women using data from antenatal care centres in Tanzania: a population-based study. Lancet Glob. Health.

[19] Stratton S.J. (2021). Population research: convenience sampling strategies. Prehosp. Disaster Med..

[20] Ehrlich H.Y. (2020). Molecular surveillance of antimalarial partner drug resistance in sub-Saharan Africa: a spatial-temporal evidence mapping study. Lancet Microbe.

[21] Nsanzabana C. (2019). Strengthening surveillance systems for malaria elimination by integrating molecular and genomic data. Trop. Med. Infect. Dis..

[22] WHO (2022).

[23] WHO (2020).

[24] Gupta H. (2017). Molecular surveillance of pfhrp2 and pfhrp3 deletions in *Plasmodium falciparum* isolates from Mozambique. Malar. J..

[25] Gamboa D. (2010). A large proportion of *P. falciparum* isolates in the Amazon region of Peru lack pfhrp2 and pfhrp3: implications for malaria rapid diagnostic tests. PLoS One.

[26] Agaba B.B. (2019). Systematic review of the status of pfhrp2 and pfhrp3 gene deletion, approaches and methods used for its estimation and reporting in *Plasmodium falciparum* populations in Africa: review of published studies 2010-2019. Malar. J..

[27] Rogier E. (2022). *Plasmodium falciparum* pfhrp2 and pfhrp3 gene deletions from persons with symptomatic malaria infection in Ethiopia, Kenya, Madagascar, and Rwanda. Emerg. Infect. Dis..

[28] Plowe C.V. (2015). Vaccine-resistant malaria. N. Engl. J. Med..

[29] Ikeda M. (2018). Artemisinin-resistant *Plasmodium falciparum* with high survival rates, Uganda, 2014-2016. Emerg. Infect. Dis..

[30] Straimer J. (2022). High prevalence of *Plasmodium falciparum* K13 mutations in Rwanda is associated with slow parasite clearance after treatment with artemether-lumefantrine. J. Infect. Dis..

[31] Rasmussen C. (2022). Current and emerging strategies to combat antimalarial resistance. Expert Rev. Anti Infect. Ther..

[32] Diez Benavente E. (2020). A molecular barcode to inform the geographical origin and transmission dynamics of *Plasmodium vivax* malaria. PLoS Genet..

[33] Obaldia N. (2015). Clonal outbreak of *Plasmodium falciparum* infection in eastern Panama. J. Infect. Dis..

[34] de Oliveira T.C. (2020). Population genomics reveals the expansion of highly inbred *Plasmodium vivax* lineages in the main malaria hotspot of Brazil. PLoS Negl. Trop. Dis..

[35] Sy M. (2021). Genomic investigation of atypical malaria cases in Kanel, northern Senegal. Malar. J..

[36] WHO (2022).

[37] Galatas B. (2016). Malaria parasites in the asymptomatic: looking for the hay in the haystack. Trends Parasitol..

[38] Early A.M. (2019). Detection of low-density *Plasmodium falciparum* infections using amplicon deep sequencing. Malar. J..

[39] Pujol A. (2023). Detecting temporal and spatial malaria patterns from first antenatal care visits. Res. Sq..

[40] Gonzalez R. (2012). HIV and malaria interactions: where do we stand?. Expert Rev. Anti Infect. Ther..

[41] Yobi D.M. (2022). Biennial surveillance of *Plasmodium falciparum* anti-malarial drug resistance markers in Democratic Republic of Congo, 2017 and 2019. BMC Infect. Dis..

[42] Verity R. (2020). The impact of antimalarial resistance on the genetic structure of *Plasmodium falciparum* in the DRC. Nat. Commun..

[43] Guillot C. (2022). Criteria for selecting sentinel unit locations in a surveillance system for vector-borne disease: a decision tool. Front. Public Health.

[44] WHO (2009).

[45] Racloz V. (2006). Sentinel surveillance systems with special focus on vector-borne diseases. Anim. Health Res. Rev..

[46] Blickenstorfer S. (2011). Using scenario tree modelling for targeted herd sampling to substantiate freedom from disease. BMC Vet. Res..

[47] Cameron A.R. (2012). The consequences of risk-based surveillance: developing output-based standards for surveillance to demonstrate freedom from disease. Prev. Vet. Med..

[48] Adjah J. (2018). Seasonal variations in *Plasmodium falciparum* genetic diversity and multiplicity of infection in asymptomatic children living in southern Ghana. BMC Infect. Dis..

[49] Kobbe R. (2006). Seasonal variation and high multiplicity of first *Plasmodium falciparum* infections in children from a holoendemic area in Ghana, West Africa. Tropical Med. Int. Health.

[50] Mayor A. (2008). Molecular markers of resistance to sulfadoxine-pyrimethamine during intermittent preventive treatment for malaria in Mozambican infants. J. Infect. Dis..

[51] Menendez C. (2011). HIV and placental infection modulate the appearance of drug-resistant *Plasmodium falciparum* in pregnant women who receive intermittent preventive treatment. Clin. Infect. Dis..

[52] Brown T. (2012). Molecular surveillance for drug-resistant *Plasmodium falciparum* in clinical and subclinical populations from three border regions of Burma/Myanmar: cross-sectional data and a systematic review of resistance studies. Malar. J..

[53] Sy M. (2022). *Plasmodium falciparum* genomic surveillance reveals spatial and temporal trends, association of genetic and physical distance, and household clustering. Sci. Rep..

[54] Kabaghe A.N. (2017). Adaptive geostatistical sampling enables efficient identification of malaria hotspots in repeated cross-sectional surveys in rural Malawi. PLoS One.

[55] Graubard B.I., Korn E.L. (1996). Modelling the sampling design in the analysis of health surveys. Stat. Methods Med. Res..

[56] Schaffner S.F. (2018). hmmIBD: software to infer pairwise identity by descent between haploid genotypes. Malar. J..

[57] Gerlovina I. (2022). Dcifer: an IBD-based method to calculate genetic distance between polyclonal infections. Genetics.

[58] Daniels R.F. (2015). Modeling malaria genomics reveals transmission decline and rebound in Senegal. Proc. Natl. Acad. Sci. U. S. A..

[59] Lee A. (2021). Modeling the levels, trends, and connectivity of malaria transmission using genomic data from a health facility in Thiès, Senegal. medRxiv.

[60] Nkhoma S.C. (2013). Population genetic correlates of declining transmission in a human pathogen. Mol. Ecol..

[61] Searle K.M. (2017). Distinct parasite populations infect individuals identified through passive and active case detection in a region of declining malaria transmission in southern Zambia. Malar. J..

[62] Sisya T.J. (2015). Subtle changes in *Plasmodium falciparum* infection complexity following enhanced intervention in Malawi. Acta Trop..

[63] Neafsey D.E., Volkman S.K. (2017). Malaria genomics in the era of eradication. Cold Spring Harb. Perspect. Med..

[64] Nelson C.S. (2019). High-resolution micro-epidemiology of parasite spatial and temporal dynamics in a high malaria transmission setting in Kenya. Nat. Commun..

[65] Zhu L. (2018). The origins of malaria artemisinin resistance defined by a genetic and transcriptomic background. Nat. Commun..

[66] Battle K.E. (2016). Treatment-seeking rates in malaria endemic countries. Malar. J..

[67] Blasco B. (2017). Antimalarial drug resistance: linking *Plasmodium falciparum* parasite biology to the clinic. Nat. Med..

[68] Bushman M. (2018). Within-host competition can delay evolution of drug resistance in malaria. PLoS Biol..

[69] Griffith G.J. (2020). Collider bias undermines our understanding of COVID-19 disease risk and severity. Nat. Commun..

[70] Zejda J.E. (2021). Seroprevalence of Anti-SARS-CoV-2 antibodies in a random sample of inhabitants of the Katowice Region, Poland. Int. J. Environ. Res. Public Health.

[71] Lemmen S.W. (2001). Implementing and evaluating a rotating surveillance system and infection control guidelines in 4 intensive care units. Am. J. Infect. Control.

[72] Murato Y. (2020). Evaluation of sampling methods for effective detection of infected pig farms during a disease outbreak. PLoS One.

[73] Thompson S.K. (1997). Spatial sampling. CIBA Found. Symp..

[74] Salehi M., Smith D.R. (2021). Adaptive two-stage inverse sampling design to estimate density, abundance, and occupancy of rare and clustered populations. PLoS One.

[75] Case B.K.M. (2022). Spatial epidemiology and adaptive targeted sampling to manage the Chagas disease vector *Triatoma dimidiata*. PLoS Negl. Trop. Dis..

[76] Andrade-Pacheco R. (2020). Finding hotspots: development of an adaptive spatial sampling approach. Sci. Rep..

[77] Wohl S. (2023). Sample size calculations for pathogen variant surveillance in the presence of biological and systematic biases. Cell Rep. Med..

[78] Myatt M., Bennett D.E. (2008). A novel sequential sampling technique for the surveillance of transmitted HIV drug resistance by cross-sectional survey for use in low resource settings. Antivir. Ther..

[79] Ginting F. (2019). Rethinking antimicrobial resistance surveillance: a role for lot quality assurance sampling. Am. J. Epidemiol..

[80] Lanata C.F., Black R.E. (1991). Lot quality assurance sampling techniques in health surveys in developing countries: advantages and current constraints. World Health Stat. Q..

[81] Hedt B.L. (2012). Multidrug resistance among new tuberculosis cases: detecting local variation through lot quality-assurance sampling. Epidemiology.

[82] Smith-Aguasca R. (2019). Mosquitoes as a feasible sentinel group for anti-malarial resistance surveillance by next generation sequencing of *Plasmodium falciparum*. Malar. J..

[83] Nkemngo F.N. (2022). Xeno-monitoring of molecular drivers of artemisinin and partner drug resistance in *P. falciparum* populations in malaria vectors across Cameroon. Gene.

